# Inaugural Presidential Address to the Atlanta Society of Medicine

**Published:** 1904-02

**Authors:** Alex W. Stirling

**Affiliations:** M.D., C.M. (Edin.); D. P. H. (Lond.) Atlanta, Ga.


ATLANTA

### Journal-Record of Medicine.

Successor to Atlanta Medical and Surgical Journal, Established 1855, and Southern Medical Record, Established 1870.

Vol. V. FEBRUARY, 1904. No. 11.

BERNARD WOLFF, M.D., EDITOR, Nos. 319–20 Prudential.

M. B. HUTCHINS, M.D., BUSINESS MANAGER, 1007–1008 Century Bldg.

Published Monthly.

### ORIGINAL COMMUNICATIONS.

### INAUGURAL PRESIDENTIAL ADDRESS TO THE ATLANTA SOCIETY OF MEDICINE.

By ALEX W. STIRLING, M.D., C.M. (Edin.); D. P. H. (Lond.), Atlanta, Ga.

Gentlemen: When I sat down to write a few words of thanks for the honor which you have been kind enough to bestow upon me in making me your president for 1904, and to put into shape one or two propositions having in view the enhancement of the value of this Society, my mind jumped back to my boyish days among the hills and burns of a land which no native can ever hold anything but dear, but whose chief export, for all that, is the commodity called men and women. There I, like many another of my tribe, cast longing eyes towards the mysterious West, the land of the pathless wood and hardly trodden plain, the land of guns and lassoes, bear and antelope, shirts and no collars, trousers and no suspenders, fresh air, light, and freedom from restraint. Distance lent enchantment to the view. Since then I have been on the eve of seeing many parts of the globe, and have seen a few. I have grown older, considerably, and *experientia stultos docet.* I like a comfortable armchair, and I would rather put rice and potatoes than a rope on the most peaceable steer I ever saw. *Hic transit gloria mundi.*

I feel that unless you are exceedingly well behaved I shall cut a sorry figure in your presidential chair, and yet for comfort I would sooner sit upon it now than in the saddle of a bucking broncho. Which would bring me to the higher elevation is a question; but I would rather fall back upon your generous good nature than in the softest puddle in all Texas.

I have other reminiscences which might be made of more interest to you had I a kind of ability which indeed I sadly lack. I remember my first visit to the old infirmary in Edinburgh, and it was a *mauvais quart d'heure* when, escorted by a school friend who had already passed into a stage of learning which then appeared to me quite awesome, I beheld a knowing old professor with perfect calmness palpate a fearful tumor upon the head of an ancient dame, who, in my estimation, was about to enter through the gruesome gates of death. The wen, for as such I now recognize it, accordingly extracted so much of the available stock of sympathy within my green young heart that it failed completely and nearly landed me collapsed upon the floor.

I remember many famous men during my years at the university, some of whom I fear I did not appreciate. Among the most interesting scenes to you, perhaps, would be the wards of Mr. Joseph Bell. There he sat, towel on knee, his clean-cut Roman face glowing with intellect—yes, and with humor, though you will hardly believe it, as he is a Scotsman—informing us concerning the private history of each new patient presented to his view, with at first never a question on the subject. Why did not I invent Sherlock Holmes? The following year when Conan Doyle followed in my footsteps and drank in inspiration for his famous stories, Mr. Bell was just the same. There was a difference somewhere, but I can not make it out.

At this point I wish to make an interpolation. This address, if it can be dignified by such a fine name, has been written in penny numbers, during the fag ends of my time, which has been very fully occupied since I became aware of my duty in this matter. What I have just read to you was written I suppose a week ago. I subscribe to *Collier's Weekly*, and I can highly recommend it. The number for January 9th arrived to-day, and I opened it to read on the car this afternoon. There I saw the heading, "The Original of Sherlock Holmes." The article is not long, and it is both interesting and amusing. Shall I read it to you?

. . . . . . . . .

You see in this article Sir Henry Littlejohn (his name is not correctly spelled in it) is credited with a part in the making of Sherlock Holmes. I did not know that before, but I can well believe it. I spent a summer in daily contact with Sir Henry, in classes and in wandering about examining abattoirs and nuisances. He was the sharpest little ferret that ever poked his nose into any difficulty. In walk and conversation he seemed to be made of steel springs. As a raconteur he was inimitable. He drove home every point in jurisprudence and public health with an anecdote, not always, I regret to say, fit for the ears of a young ladies' boarding-school; and though then he was only an extra-mural lecturer, the classes of the likewise famous professor of these subjects in the university and himself a well-known wit and singer of his own medical canticles, Sir Douglas MacLagan, suffered considerably from the hilarity of Littlejohn's rival establishment. His wit was of the keenest, and he handled it with as much dexterity as the most experienced barber does his razor.

And I hope, gentlemen, that I am not stretching your credulity beyond the breaking-point, for Sir Henry, too, was a Scotsman of the Scots. But it is high time to return to the address at the point at which I left it.

Instead of watching the unconscious Sherlock Holmes, would you rather stand in the big operating theater, crowded to the roof, and wish you were Isaac Bayley Balfour, later professor of botany in Oxford, and now promoted into the chair of his father in Edinburgh? He was then only a medical student, and had been selected to read the address to one, Joseph Lister, then professor of clinical surgery in Edinburgh, and recently in receipt of a pressing invitation to forsake the cold, gray capital of the north for the allurements of the metropolis of the world. When the tall and stately form did enter, you too would have joined in the shout which rent the air, tore it into fragments, and continued minute after minute till his face, the embodiment of refinement, now so well known to all the world, softened and then quivered, till his outstretched arm requested silence. You too would have welcomed the absolute stillness which pervaded that crowded amphitheater, when with low and shaking voice the man who in those days has perhaps done most for the happiness of the human race, that inverted Napoleon, told us that the ties which bound him to us were too strong, he thought, for breaking.

Yet he broke them, and probably he was right. But with him he took to London a band of Edinburgh students to teach his methods to the world.

But I have said sufficient in this strain and it is time for business.

I wish first to thank you for having made me your president. It is an honor; and now that I occupy the position, I feel that it entails no inconsiderable responsibility, so much so that I wish I were better fitted to fill it. I feel especially that the pleasing, urbane manners of my immediate predecessor put me in an awkward fix. But after a rose last year you should be better prepared to stand a thistle this.

It is a position of responsibility, because a society of this nature may have an influence which reaches further than we are apt to notice at a glance. A hint dropped here may in reality be a matter of life or death for patients, with honor or dishonor for ourselves. While it would be quite untrue to say that familiarity with the anguish of others breeds contempt for it, it may contain a particle of truth to say that the monotony of "the daily round, the common task," and the weariness which must needs follow on anxiety and constant application, dull our minds perhaps a little to the breadth and the height and the depth of the trust which is committed to us by reason of our profession, so that we forget to be greedy of knowledge and fail to do our very utmost for those who in the nature of things must almost blindly rely upon our honor and our ability to help them.

In order as far as I am able to continue in the path of progress trodden by my predecessors, I have been considering what might be done to increase the usefulness of the Society. It is apparent that the great object to be aimed at is the increase of knowledge, but that is not all—we must aim also to stimulate enthusiasm for professional work, to cultivate high ideals, and to augment the pleasant relationship which already exists in our midst, I think in a notable degree. With a view to forwarding such ends I bring before you the following propositions.

But before proceeding to these I wish to make a remark. It has long appeared to me that nowhere does the medical profession take that part which it ought to take in moulding public opinion on matters with which it alone is conversant. I do not wish to make any proposition in this connection, but I do wish to say that I should be glad to see the matter ventilated among us. For instance, I think we ought to try harder to impress the public with certain laws of health preservation—the prevention of disease. I have often thought that had I the means I would employ a committee of experts to write a pamphlet on the subject, keep it up-to-date, and see that each household had one by, for instance, presenting it on the registration of a marriage or a birth. There is the deadly poisoning of children by so-called food, and there are other subjects equally important. In some cities of my acquaintance lectures are given for the people. They are free, and the printed lecture is afterwards sold for two cents a copy. A matter in which we should be interested is one which is a sore subject with us, funerals. When one thinks of the process of decay of the body after the ordinary burial the mind turns away disgusted. There is a kind of coffin which provides the real earth-to-earth burial, in which the antiseptic action of the ground is allowed full play. Where there is no cremation this should be preferred, and a statement on the subject from a medical society, no names appearing, would go a long way with the public.

But to come to the propositions:

1. I ask you to approve the action which I have already taken in regard to the paper for 1904. I chose a number of subjects which appeared likely to be generally interesting, and invited several members of the society to meet and consider these, as well as the members who should be asked to discuss them. With their assistance these were arranged and rearranged and new subjects were suggested, and I can now lay before the society a programme for the year, consisting, in most cases, of at least two papers for each evening. Those members whose names are there mentioned have all promised to take the parts allotted to them. In this way it is hoped to make sure of an interesting debate by prepared speakers for each meeting. The programmes I ask you to agree to have immediately printed, to have upon them also certain extracts from the regulations of this society and certain information concerning its workings. I have here the programme as I should like to see it printed, and I can read it now or preferably later when we discuss each proposition. These should be made suitable for hanging upon our desks. I do not mean to suggest that the programmes are to relieve our secretary of the duty of sending out post-cards before each meeting as is now done. No, he must work for such a salary as his; and I would also suggest that the post-cards reach the members the day before the meetings.

I desire before leaving this subject to say that because the names of certain members are placed upon this programme there is of course no intention to interfere with free discussion. On the contrary I have done my best to enlarge the number of speakers, but with some members, after repeated attempts by telephone, I have failed to get into communication. Some have preferred to act the important part of listener, and time has been too limited to admit of addressing every member as I intended. I hope that any members present who care to be put on the list will give in their names to-night.

2. There are a number of men in Atlanta who should be in the society and are not. The secretary has a list of the profession here. I suggest that this be gone over, and an endeavor be made to enlarge the attendance.

3. The most interesting and instructive method of gaining medical knowledge is undoubtedly by the observation of cases. There is room for improvement in this respect in our Society. Let us fill this vacancy. We should have, I think, at least two cases at every meeting, and if you will permit me I shall appoint a committee of twelve to provide these cases, each from his own practice or that of his friends. But, as one man usually does all the work of a committee, I shall also suggest, in order to prevent this, that members of the committee provide the cases in alphabetical order, the chairman's duty being to see that each member does his part. The names of this committee I have ready in case you approve of this suggestion. Any member having a case to show would be requested to communicate with the chairman of the committee, or with Dr. Emery, the Society's secretary. The nature of the case and the names of the demonstrators would be put upon the cards sent out before each meeting. I do not mean that each member of the committee must necessarily himself show the cases for his evening. I mean that he would be responsible for the production of two demonstrations by some one.

I would also suggest, for the sake of the patients presenting themselves, that they be considered after the application for membership, and that a certain time only be allotted to them. For this reason it would be well that the patients should be present by eight o'clock, and that we be a little more punctual than in the past; and I would suggest that the meetings be called to order at eight o'clock. The ordinary business would then be proceeded with not later than a definite time, say at 8:45, but by the consent of a majority of the members present, obtained by formal motion made and carried, further time might be occupied with the cases.

4. It has been suggested to me that distinguished men from other parts of the country be from time to time invited to read papers before us.

There are two sides to this question, and I should prefer to have the opinion of the Society before taking any steps in this direction.

5. It has also been suggested to me that the Society offer a prize of some description to be competed for annually by theses, and to be named after some famous Southern doctor. I have pleasure in laying this suggestion before you for consideration.

6. A suggestion was brought forward, I think, at our last annual meeting, and highly approved, that the medical students at the college be invited to attend these meetings without fee. I do not know that anything practical resulted. With your permission I will request one of the members to be good enough to take upon himself the responsibility of keeping a permanent notice in a conspicuous place at the college, and also to see that the cases, subjects for discussion, and names of those taking part, be put beside it at least one day before each meeting. With your approval I will name Dr. T. F. Harris and ask him to do this himself, or to see that it is done. I may say he has already consented to undertake this duty if you approve it. I would also suggest that the military members of the profession in the vicinity and the resident house physicians and house surgeons of the Grady Hospital be invited to take part in our discussions as visitors, without voting powers, unless they prefer to join as ordinary members.

7. There is the ever-recurring question of how we are to deal with those who are behind in paying their dues. I have obtained from the treasurer a list of twenty-eight members who are in this position, representing a sum of $304.00. I suggest that we discuss the proper steps to be taken in this important matter.

8. There are sure to be good things said during the year which will go unrecorded unless we have a stenographer. What are we to do to get one? As a good medical stenographer is hard to find, and as, if found, we could not pay him properly, I suggest that each speaker be kind enough to send the following day in writing to the secretary the substance of his remarks.

9. The time of the meeting of the State Association coincides with ours of April 21st. I propose that we meet instead on April 28th. Also, last summer the meetings were so pleasant that by special motion they were carried through July and August without interruption. In order to accommodate the papers promised, and in the hope that this year a like desire would be manifested not to break into the series, I have filled up the programme for these months. I shall be glad if you will say what you desire to do on the subject.

10. It has become almost a custom among certain members of the Society to have a social gathering after the meeting. Club sandwiches, celery, etc., and beer or coffee and cigars, have usually been the fare, and in my experience members have generally had to wait some considerable time for service, and the charges have amounted to about 75 cents each. This is not a large sum, but when frequently repeated it is more than some members may care to spend for such a purpose. In order to make these social gatherings a matter of less moment to the pocket, as well as the digestion, and to increase their popularity, I have seen Mr. Lee Barnes, of the Aragon Hotel, and he is willing to provide for 25 cents each a bottle of beer or a cup of coffee and crackers and cheese, *q. s.*, to stave off the pangs of hunger and give something to nibble at, these being ready for us when we adjourn from the library. Any one desiring more would be required to pay extra for it. During the course of the evening it would be necessary to send to the hotel the number of those who were to be expected there. I have consulted several members who have been accustomed to join these meetings in the past, and it is only because the idea has their approval that I make this suggestion.

I have arranged nothing for this evening, and if there is a disposition to meet after adjournment, perhaps one of our members would kindly discover the number of those who will be present, decide what fare is desired, and send word to Mr. Barnes.

11. There is a last matter which I wish to bring to your attention. It is not exactly a matter appertaining to this Society at all, but the true welfare of the Society is inseparable from it—I mean the Medical Library. Beyond being a member of the Library Committee I have no special authority to speak on the subject, but it is with the hearty approval of the president of the Library, Dr. Hoke, and its secretary, Dr. Block, that I urge members to put into the Library the price of one journal and get back fifty. Atlanta should be the medical center of all the surrounding States, and I once brought forward a scheme by which to include non-residents among its readers, and had thought of doing so again when the time seemed ripe. But we can not possibly invite outsiders to support the movement so long as those upon the spot are not efficiently carrying out their part.

Arrangements are being made to give us a comfortable reading-room, with protection for the journals.

I beg again to thank you for the confidence you have reposed in me, and to wish for the Society and each member a happy and prosperous year.

